# Examining the Post-Discharge Effects of Supportive Text Messaging and Peer Support: No Significant Improvements in Mental Health Recovery or Quality of Life

**DOI:** 10.1192/j.eurpsy.2026.11345

**Published:** 2026-07-31

**Authors:** E. Owusu, W. Mao, R. Shalaby, H. E. Elgendy, B. Agyapong, E. Eboreime, M. A. Lawal, N. Nnamdi, P. Chue, A. J. Greenshaw, V. I. O. Agyapong

**Affiliations:** 1 Psychiatry, University of Alberta, Edmonton; 2 Psychiatry, Dalhousie University, Halifax, Canada

## Abstract

**Introduction:**

The transition from psychiatric hospitalization to community living is often marked by stress and emotional challenges. discharged from acute mental health facilities in Alberta.

**Objectives:**

This study examined the impact of supportive text messages (Text4Support) and peer support services (PSS) on resilience, personal recovery, and quality of life in individuals

**Methods:**

Participants (N = 1,098) were allocated to three groups: Treatment as Usual (TAU, 40%), Supportive Text Messages (SMS, 49.3%), and Peer Support + SMS (PSS, 10.7%). Outcome measures included the Brief Resilience Scale (BRS), Recovery Assessment Scale (RAS), and Quality-of-Life assessments, analyzed using chi-square tests, one-way ANOVA, and ANCOVA.

**Results:**

Only 34% completed at least one follow-up, with 15% completing the 6-month evaluation. No significant differences were found between the TAU and intervention groups at the 6-month mark.

**Conclusions:**

These findings suggest limited evidence for the effectiveness of SMS or PS in improving outcomes over six months, though the low follow-up rate may limit generalizability. Future research should explore the conditions that optimize intervention effectiveness and examine differences between respondents and non-respondents to assess potential long-term benefits.

**Disclosure of Interest:**

None Declared

